# Supplementary material for: In Silico and In Vitro Structure–Activity Relationship of Mastoparan and Its Analogs
Source: Molecules. 2022 Jan 16;27(2):561. doi: 10.3390/molecules27020561 (PMC8779355; doi:10.3390/molecules27020561)
Supplement: Supplementary file 1 [file molecules-27-00561-s001.zip › molecules-1525857-supplementary.pdf]

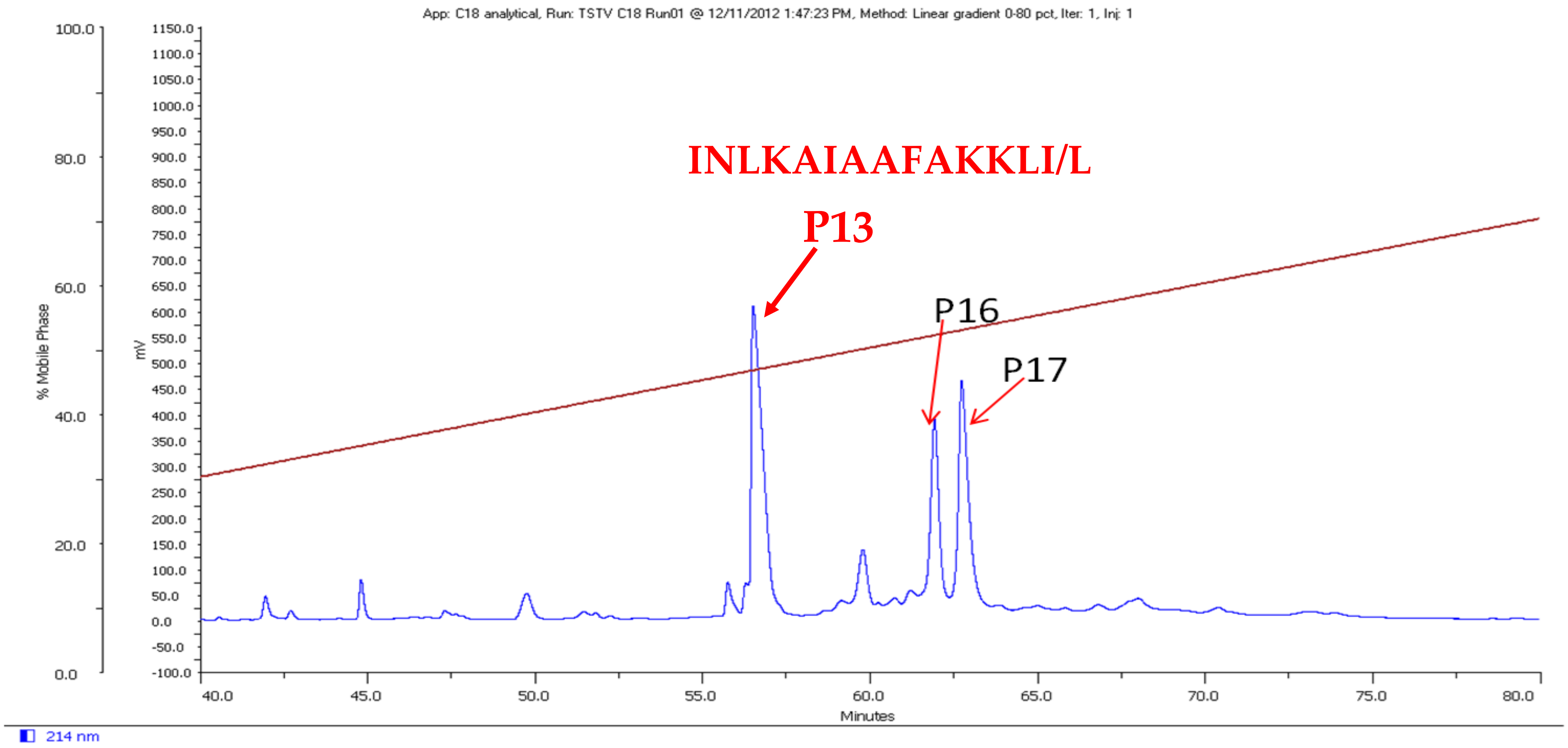

Figure S1. Purification profile using HPLC-C18 column. Soluble *V. tropica* venom was loaded onto a C18 reverse phase HPLC column. A linear gradient of 0% solvent A (0.1% trifluoroacetic acid (TFA) in water), and 80% solvent B (0.085% TFA in acetonitrile) were run for 100 min at a flow rate of 1 mL/min. The absorbance was monitored at 214 and 280 nm. The purification revealed 21 fractions. The fraction P16 and P17 exhibited phospholipase activity [19]. Only P13 showed antibacterial activity, the N-terminal sequences were "INLKAIAAFAKKLI/L" which is mastoparan peptides.
